# Terahertz Metamaterial with Multiple Resonances for Biosensing Application

**DOI:** 10.3390/nano10061038

**Published:** 2020-05-29

**Authors:** Huiliang Ou, Fangyuan Lu, Zefeng Xu, Yu-Sheng Lin

**Affiliations:** State Key Laboratory of Optoelectronic Materials and Technologies, School of Electronics and Information Technology, Sun Yat-Sen University, Guangzhou 510275, China; ouhliang@mail2.sysu.edu.cn (H.O.); lufy@mail2.sysu.edu.cn (F.L.); xuzf6@mail2.sysu.edu.cn (Z.X.)

**Keywords:** metamaterial, multiple resonances, biochemical sensing, environment sensor

## Abstract

A sickle-shaped metamaterial (SSM) based biochemical sensor with multiple resonances was investigated in the terahertz frequency range. The electromagnetic responses of SSM were found to be four resonances, namely dipolar, quadrupolar, octupolar and hexadecapolar plasmon resonances. They were generated from the interactions between SSM and perpendicularly incident terahertz waves. The sensing performances of SSM-based biochemical sensors were evaluated by changing ambient environments and analyte varieties. The highest values of sensitivity and figure of merit (FOM) for SSM covered with analyte thin-films were 471 GHz/RIU (refraction index unit) and 94 RIU^−1^, respectively. In order to further investigate the biosensing ability of the proposed SSM device, dielectric hemispheres and microfluidic chips were adopted to imitate dry and hydrous biological specimens, respectively. The results show that the sensing abilities of SSM-based biochemical sensors could be enhanced by increasing either the number of hemispheres or the channel width of the microfluidic chip. The highest sensitivity was 405 GHz/RIU for SSM integrated with microfluidic chips. Finally, three more realistic models were simulated to imitate real sensing situations, and the corresponding highest sensitivity was 502 GHz/RIU. The proposed SSM device paves the way to possible uses in biochemical sensing applications.

## 1. Introduction

Recently, terahertz spectroscopy has attracted great interest due to its unique characteristics and availability in massively promising applications [1,2,3]. It has many photoelectric characteristics, such as low photon energy but high penetration, as well as non-contact and label-free inspection, which enable terahertz spectroscopy technology to detect chemicals and biomolecules [4,5]. In addition, terahertz waves are consistent with the inherent frequency of some important biomarkers, such as nucleic acid and specific proteins, which make terahertz biochemical sensors possible [6,7,8]. However, the optical properties of natural materials are inherent and cannot be changed. Moreover, terahertz waves’ transmission is high for common plastics and fibers, and their reflectivity is also high for most metal materials. These optical properties represent a bottleneck for the interaction between natural materials and terahertz waves [9]. Metamaterial is a composite material that possesses unique electromagnetic properties realized by the configuration of specific structures. Near-field electromagnetic energy can interact and then be enhanced within the metamaterial. The combination of terahertz waves and metamaterial means that the incident electromagnetic wave can be controlled and manipulated. 

The configuration of a metamaterial is a kind of synthetic subwavelength array structure which features negative permittivity, permeability, perfect absorption and superlens capability [10,11]. Metamaterial can be designed to possess single, dual, triple and multiple resonances by tailoring diversified metamaterial patterns and structures [12,13,14]. Among these plasmonic resonances, the frequency shift of higher-order plasmonic resonances with high quality factors (Q-factor) is ultrasensitive to the geometrical shapes and the local dielectric environment [15,16]. Therefore, plasmonic metamaterial-based sensors are usually compact, portable and cost-effective, possessing great potential for detecting different kinds of chemicals as well as biomolecules in trace amounts [17,18,19]. The utilization of plasmonic metamaterial-based sensors with high efficiency and high Q-factors is the essential reason for developing an ultrahigh-sensitivity biosensor. 

In this study, a plasmonic metamaterial-based biochemical sensor with multiple resonances and high sensitivity is presented in the terahertz frequency range. The proposed plasmonic metamaterial is composed of an array of a pair of centrosymmetric sickle-shaped metamaterials (SSM). The ambient environments and analyte varieties were changed to investigate the sensitivity of SSM-based biochemical sensors. The higher-order plasmonic resonance was accompanied by a larger red-shifting of the resonance. The highest sensitivity was 471 GHz/RIU (refraction index unit) for the hexadecapolar plasmonic mode of SSM. The corresponding figure of merit (FOM) was 94 RIU^−1^. We further investigated the influences of dielectric hemispheres and microfluidic chips to imitate the dry and hydrous biological specimens. The results show that the red-shifting of the resonance could be increased by raising the number of hemispheres and the channel width of the microfluidic chip. The relationships between resonance shifting, hemisphere quantity and the channel width of the microfluidic chip are quite linear. The proposed SSM device provides an effective approach to detecting and analyzing the chemicals and biomolecules. 

## 2. Designs and Methods

**Figure 1:**
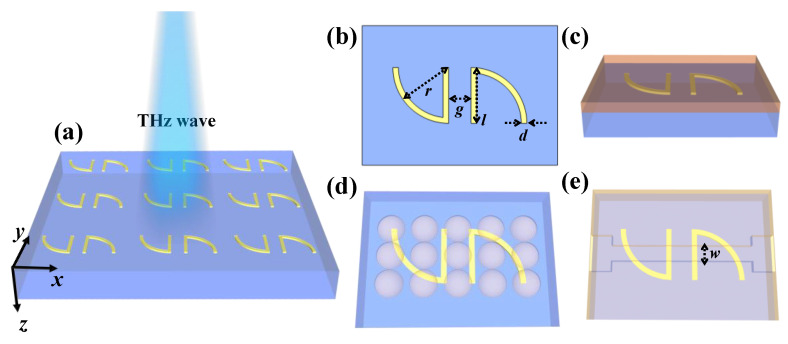
(**a**) Schematic drawing of the sickle-shaped metamaterial (SSM). (**b**) Geometrical denotations for the corresponding SSM unit cell. (**c**–**e**) Schematic drawings of SSM covered with (**c**) analyte thin-film, (**d**) dielectric hemispheres and (**e**) microfluidic channels.

Figure 1a shows a schematic drawing of the periodic SSM structure. The incident terahertz wave propagates perpendicularly to the SSM surface along the *z*-axis. A schematic diagram of an SSM unit cell is illustrated in Figure 1b, which includes the geometrical denotations. The SSM unit cell is composed of a pair of centrosymmetric Au split-ring resonators (SRRs) on a polydimethylsiloxane (PDMS) substrate. The permittivity of the Au material is described using the Drude model as expressed by [20,21,22,23]:

(1) $$\varepsilon (\omega )=1-\frac{{\omega_{p}}^{2}}{\omega [(\omega +i\omega_{c})]}$$

where $\omega_{p}$ = 1.37 × 10^16^ Hz is the plasmon frequency and $\omega_{c}$ = 4.08 × 10^13^ Hz is the scattering frequency for the Au material [20,24].

**Figure 2:**
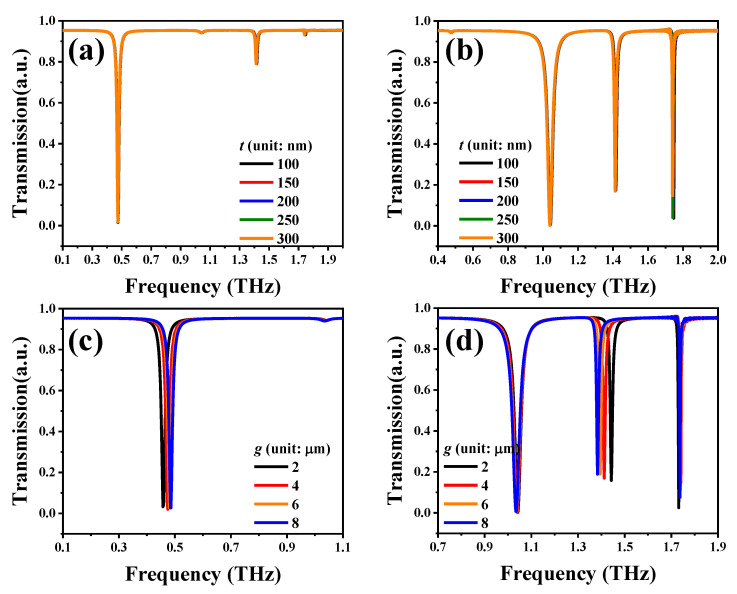
Transmission spectra of SSM with different *t* values in (**a**) transverse electric (TE)) and (**b**) transverse magnetic (TM) modes. Transmission spectra of SSM with different *g* values in (**c**) TE and (**d**) TM modes.

Figure 1c shows the SSM device covered with an analyte thin-film serving as a chemical specimen. To further investigate the sensing of dry and aqueous biological specimens, dielectric hemispheres and microfluidic channels were integrated with the SSM device, as shown in Figure 1d,e. The full-field electromagnetic waves were simulated using the finite-difference time-domain (FDTD) method. In the numerical modeling, the periodic boundary conditions are adopted in both the x-axis and y-axis directions while the perfectly matched layer (PML) boundary condition is set in the *z*-axis direction. The incident polarized waves are defined as transverse electric (TE) and transverse magnetic (TM) modes when the electric (E) field of incident terahertz wave is along the x-axis and y-axis, respectively. To optimize the geometrical structures of the proposed SSM, the transmission spectra of SSM with different gaps between two SRRs (*g*) and thicknesses of Au layers (*t*) are shown in Figure 2. Figure 2a,b shows the transmission spectra of SSM with different *t* values in TE and TM modes. The transmission spectra are almost the same and there are no impacts in TE and TM modes. Therefore, the *t* value is defined as the average value of 200 nm in this study. Figure 2c,d shows the transmission spectra of SSM with different *g* values in TE and TM modes. The resonant intensities are almost the same and there is a little shift on the resonant frequencies. Since the variations of resonant frequencies have no significant influence on the sensing performance of SSM, the gap distance between SRRs is defined as 4 μm in this study. Other geometrical parameters are defined as follows: arc radius, *r* = 20 μm; width of Au lines, *d* = 2 μm; length of metallic bar, *l* = 20 μm (Figure 1b). The SSM period is 60 × 60 μm^2^.

## 3. Results and Discussion

**Figure 3:**
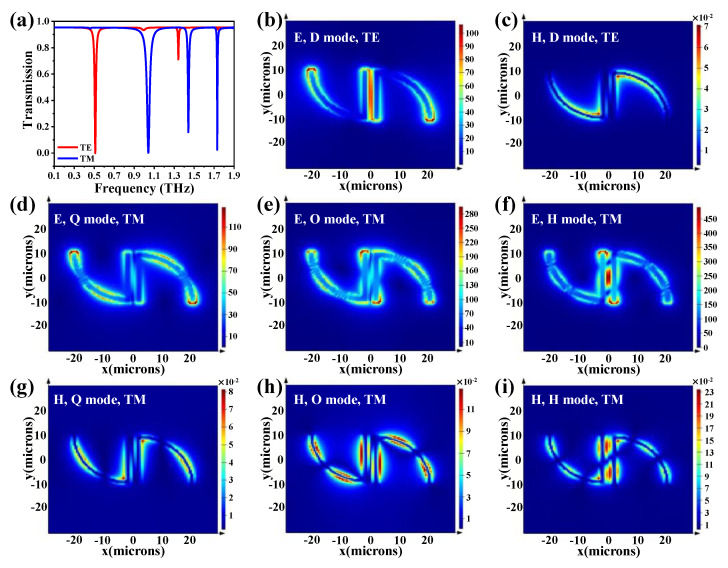
Transmission spectra of SSM and corresponding E- and H-field distributions. (**a**) Transmission spectra of SSM in TE and TM modes under the condition of *n_b_* = 1.0. (**b**) E-field and (**c**) H-field distributions of SSM monitored in resonant dipolar mode (D mode) under the incident TE-polarized wave. (**d**–**f**) E-field and (**g**–**i**) H-field distributions of SSM monitored at resonant quadrupolar mode (Q mode), octupolar mode (O mode) and hexadecapolar mode (H mode) under the incident TM-polarized wave.

Figure 3a shows SSM transmission spectra in TE and TM modes under the condition of an environmental refraction index of 1.0. The geometrical parameters are kept constant at *r* = 20 μm, *d* = 2 μm, *l* = 20 μm, *g* = 4 μm and *t* = 200 nm. Here, the environmental refraction index is denoted as *n_b_*. A single resonance is at the lower frequency of 0.51 THz for the incident TE-polarized light, as the red curve shows in Figure 3a, while there are three resonances at the higher frequencies of 1.04, 1.44 and 1.73 THz for the incident TM-polarized light, as the blue curve shows in Figure 3a. The spectrum shape of the resonance is narrower at higher resonant frequencies than at lower resonant frequencies. This is one of the prominent characteristics of multipolar plasmon modes. In addition, the higher-order plasmon mode exhibits higher sensitivity in refraction-index sensing applications. The electromagnetic-field distributions of SSM at corresponding resonant frequencies are displayed to better understand the multipolar plasmon modes. The E- and H-fields of four resonances in the sorting orders are shown in Figure 3b–i. In Figure 3b, in terms of a single SRR, the E-field energy is mainly distributed on two sides, implying that this resonance is generated from the electric dipolar mode. Actually, the order of plasmon modes (o) satisfies the equation o = *n* − 1, where *n* is the number of notes in an open SRR [12]. For instance, the *n* and o values in Figure 3b are 2 and 1, respectively, and so the resonance mode at 0.51 THz is the fundamental plasmon resonance mode, i.e., the dipolar plasmon mode (D mode). Analogously, the orders of plasmon modes in Figure 3d, Figure 3e and Figure 3f are 2, 3 and 4, respectively. This indicates that the resonance modes at 1.04, 1.44 and 1.73 THz are quadrupolar (Q), octupolar (O) and hexadecapolar (H) plasmon-resonance modes, respectively. The phenomena of different plasmon modes excited by different polarized waves could be explained in terms of structural asymmetry and phases of internal fields. The gap in a split ring leads to polarization anisotropy and allows both odd and even plasmon modes to be excited. When the symmetrical structure is broken, that can be stimulated and then generate higher-order plasmon modes, such as Q and O modes [12,25,26]. The incident TE-polarized wave results in internal fields with opposite phases on both sides of the SRR. Therefore, the E-field energy is coupled with the SRR, generating the fundamental plasmon resonance, which is the dipolar mode, as the red curve shows in Figure 3a. When the incident polarized wave is perpendicular to the split gap, i.e., the TM mode, the confined electromagnetic fields are in same phases on both sides of the SRR. The even-order plasmon modes, such as Q and H modes, are thus generated, as the blue curve shows in Figure 3a. The O mode is generated from the electromagnetic wave excited in TM mode due to the mirror symmetrical structure being broken. 

**Figure 4:**
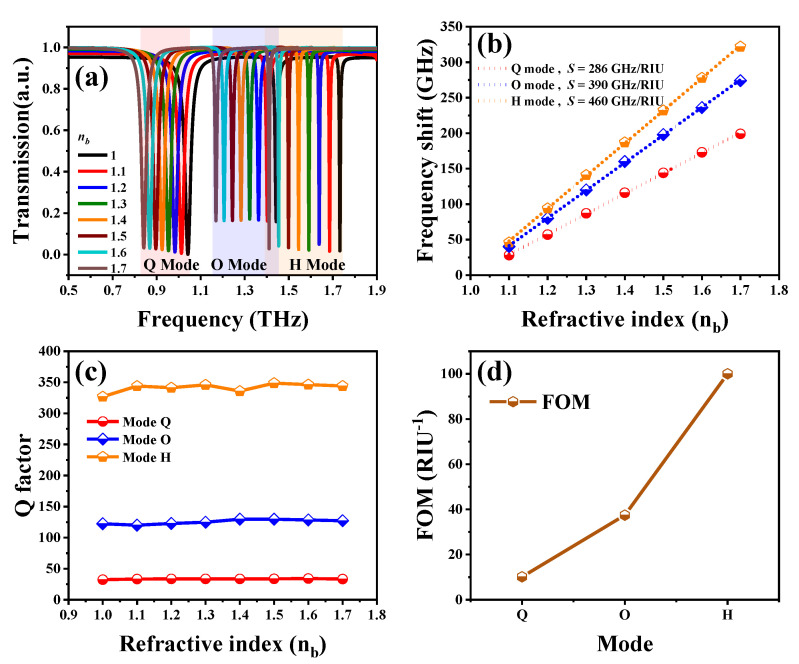
(**a**) Transmission spectra of SSM with different *n_b_* values. (**b**) Frequency shifts and (**c**) Q-factors plotted against the *n_b_* values. (**d**) The average figure of merit (FOM) values of Q, O and H modes.

The strong localized E- and H-fields emerged on the designed SSM surface, which indicated that a sharp resonance with a high Q-factor will be generated. In order to utilize the above-mentioned merits of multiple resonances in biosensing applications, we further compared and analyzed the changes of the surrounding environments and analyte varieties and then covered the SSM surface with different dielectric hemispheres and microfluidic chips to investigate its influence on sensing performance. The environmental refraction index (*n_b_*) was changed from 1.0 to 1.7 to verify the sensing ability of SSM. The transmission spectra of SSM with different *n_b_* values in TM mode are shown in Figure 4a. There are three resonances at 1.04, 1.44 and 1.73 THz for the initial condition of *n_b_* = 1.0. By increasing the *n_b_* value, the resonances are apparently red-shifted and the resonant intensities kept stable. Figure 4b plots the relationships between frequency shifts and *n_b_* values. The resonant frequencies are red-shifted linearly by increasing *n_b_* values. The shifting range of H mode is the largest compared to that of Q and O modes. The sensitivity (*S*) of an SSM is defined as the derivative of the frequency shift with respect to the refraction index, i.e., the slopes of the dashed lines shown in Figure 4b. The calculated *S* values of Q, O and H modes were 286, 390 and 460 GHz/RIU, respectively. Furthermore, when the order of the plasmon mode increases, the resonance becomes sharper and the spectrum width is narrower. Q-factor is defined as the ratio of resonant frequency to the full width at half maximum (FWHM) transmission intensity, which is used to describe the quality of each resonant frequency. In Figure 4c, Q-factors of Q, O and H modes are quite stable and insensitive to *n_b_* values. The average Q-factors are 33, 124 and 342 for Q, O and H modes, respectively. For biosensing performance, FOM is an important factor that determines the sensing abilities in the biochemical and biomolecule applications. FOM is defined by *S*/*FWMH*. The average FOM values of Q, O and H modes are 10, 37 and 100 RIU^−1^, respectively, as plotted in Figure 4d. The sensing ability of SSM could be enhanced by increasing the order of plasmon modes.

**Figure 5:**
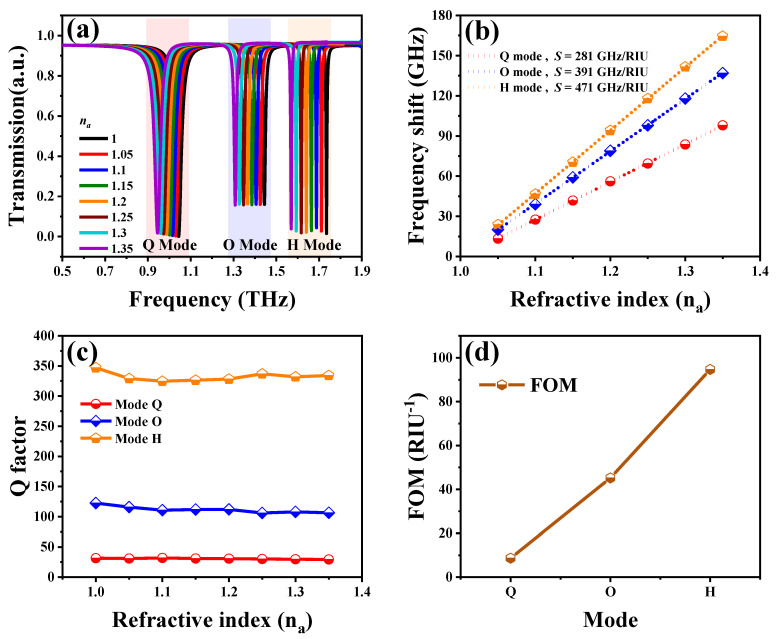
(**a**) Transmission spectra of SSM covered with different analyte thin-films. (**b**) Frequency shifts and (**c**) Q-factors plotted against the *n_a_* values. (**d**) The average FOM values of Q, O and H modes. The dielectric hemispheres were introduced to imitate the detection of cells, such as the monitoring of cell apoptosis, as shown in Figure 1d. The refraction index of dielectric hemispheres (*n_c_*) was investigated to study the electromagnetic behaviors of SSM for biosensing applications.

We also investigated the effect of SSM covered with an analyte thin-film to compare the sensing ability of SSM. The refraction index of analyte thin-film is denoted as *n_a_*. The thickness of analyte thin-film is 10 μm, as shown in Figure 1c. The transmission spectra of SSM with different *n_a_* values from 1.0 to 1.35 are shown in Figure 5a. There are three resonances red-shifted by increasing *n_a_* values. To express the red-shifts of three plasmon modes explicitly, the relationships of frequency shifts and *n_a_* values are summarized in Figure 5b. The relationships are quite linear. The frequency-shift tendency reveals that the sensitivity of H mode (471 GHz/RIU) is higher than that of O mode (391 GHz/RIU) and of Q mode (281 GHz/RIU). To further obtain the quantitative descriptions for the sensing performances, Q-factors and FOM values of plasmon modes were calculated, as shown in Figure 5c,d, respectively. The average Q-factors of Q, O and H modes were 31, 112 and 328, respectively, while the average FOM values of Q, O and H modes were 9 RIU, 45 and 94 RIU^−1^, respectively. The frequency-shift mechanism derived from the variation of *n_a_* values can be explained by the perturbation theory [5,9,27,28].

(2) $$\frac{\Delta \omega }{\omega_{0}}=\frac{\int_{\Delta V}(\mu |{\bar{H}}_{0}{|}^{2}-\varepsilon |{\bar{E}}_{0}{|}^{2})\mathrm{dv}}{\int_{v_{0}}(\varepsilon |{\bar{E}}_{0}{|}^{2}+\mu |{\bar{H}}_{0}{|}^{2})\mathrm{dv}}$$

Here, perturbation means the variation of *n_b_* values. *E*_0_ and *H*_0_ represent the unperturbed E-field and H-field, respectively. Δε ($\Delta \varepsilon =\varepsilon -\varepsilon_{0}$) and Δμ ($\Delta \mu =\mu -\mu_{0}$) are the changes to the dielectric constant and permeability after perturbation, respectively. Δω ($\Delta \omega =\omega -\omega_{0}$) is the change to the resonant angular frequency and *v*_0_ is the effective integral volume. It should be noted that Equation (2) is based on shape perturbations in terms of SSM. The perturbation is *n_b_*, with no *v*_0_. In addition, the effect of permeability on frequency shifts is negligible since the proposed SSM is non-magnetic. Therefore, it can be expressed by:

(3) $$\frac{\Delta \omega }{\omega_{0}}=\frac{-\int_{v_{0}}\Delta \varepsilon |{\bar{E}}_{0}{|}^{2}\mathrm{dv}}{\int_{v_{0}}(\varepsilon |{\bar{E}}_{0}{|}^{2}+\mu |{\bar{H}}_{0}{|}^{2})\mathrm{dv}}$$

It can be seen that the Δε value decreases by increasing the *n_b_* value according to *n_b_* = $\sqrt{\mu_{r}\varepsilon_{r}}$. Consequently, the Δω value decreases and the resonant frequency is then red-shifted.

**Figure 6:**
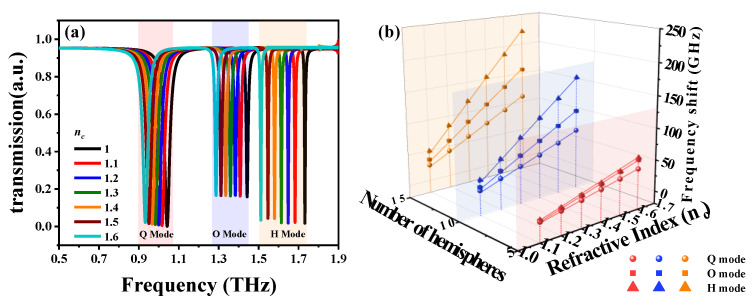
(**a**) Transmission spectra of SSM covered with different hemispheres under the condition of a hemisphere quantity of 15. (**b**) Summaries of the relationships of frequency shifts and *n_c_* values and hemisphere quantities.

Figure 6a shows the transmission spectra of SSM covered with different hemispheres under the condition of a hemisphere quantity of 15. The resonances are red-shifted by increasing *n_c_* values from 1.0 to 1.6. This indicates that the proposed SSM device has the ability to sense biological cells with different refraction indices. The relationships of *n_c_* values and frequency shifts are summarized in Figure 6b. SSM exhibits an effective frequency shift by increasing the hemisphere number or plasmon mode. For example, when the hemisphere number is fixed at 10, as the blue plane shows in Figure 6b, the resonances are red-shifted with a slope of 262 for H mode. This is larger than that of O mode (slope = 174) and of Q mode (slope = 119). Moreover, when the *n_c_* value is kept constant, the frequency shifts become larger by increasing the hemisphere number. The proposed SSM is useful for the detection of cell proliferation or cell apoptosis. When the plasmon mode is determined, the resonances are red-shifted with a slope of 359 for a hemisphere number of 15 in H mode. This is larger than that for a hemisphere number of 10 (slope = 262) and a hemisphere number of 5 (slope = 97) in H mode. The above-mentioned results indicate that the biosensing performance of SSM could be enhanced by either utilizing higher plasmon-oscillation modes or increasing the concentration of biomolecules.

**Figure 7:**
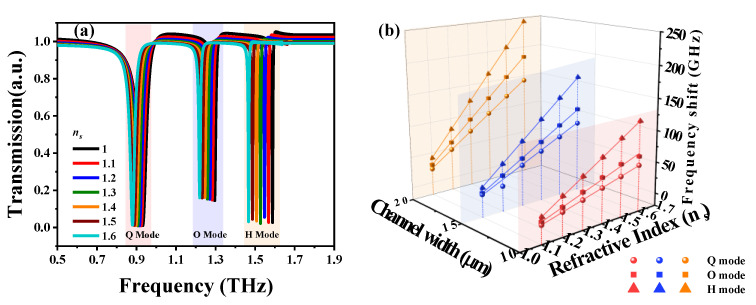
(**a**) Transmission spectra of SSM integrated with a microfluidic chip. The chemical solutions with different refraction indexes (*n_s_*) are injected into the microfluidic channel with a channel width of 10 μm. (**b**) Summaries of relationships between frequency shifts, *n_s_* values and channel widths.

We further investigated the sensing performance of SSM integrated with a microfluidic chip. This has great potential for sensing biosamples, owing to the fact that most biological specimens are dissolved in aqueous environments. Furthermore, the requirement for an injected analyte sample is micro-/nanoliters. This minute amount of analyte sample can significantly prevent the terahertz absorption of the water and thus improve the sensing capacity of SSM. The thickness and width of the microfluidic channel are 20 μm and *w*, as shown in Figure 1e. The microfluidic channel is superimposed on the split gaps of SSM, i.e., the region with strongest E-field energy. Therefore, the resonances of SSM could be tuned by injecting different chemical solutions into the microfluidic channel. Here, the refraction index for injecting chemical solutions into the microfluidic channel is denoted as *n_s_*. Figure 7a shows the transmission spectra of SSM with different *n_s_* values from 1.0 to 1.6, keeping *w* constant at 10 μm. SSM exhibits three resonances with consistently resonant intensities under the variation of *n_s_* values. These three resonances are red-shifted by increasing *n_s_* values for Q, O and H modes. The relationships between frequency shifts, *n_s_* values and *w* values are summarized in Figure 7b. The relationships are kept linear between frequency shifts and *n_s_* values by keeping the channel width constant. The resonances are red-shifted by increasing the plasmon orders. When a resonant plasmon mode is determined, the frequency shifts can be enhanced by enlarging the microfluidic channel width. Therefore, the highest sensitivity of an SSM integrated with microfluidic chip emerged in H mode when the channel width was 20 μm; this was 405 GHz/RIU. These results imply that the SSM device is ultrasensitive to the variation of the surrounding environment and thus is a potential platform for biosensing biological specimens.

**Figure 8:**
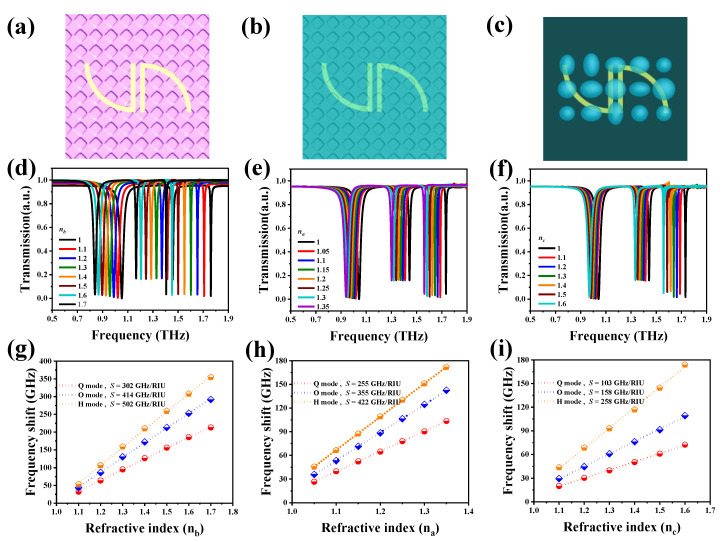
Schematics of top views of SSM (**a**) on a rough polydimethylsiloxane (PDMS) substrate, (**b**) covered with a crude analyte thin-film and (**c**) covered with random ellipse particles. (**d**–**f**) Transmission spectra of SSM (**d**) on rough PDMS substrate with different *n_b_* values, (**e**) covered with different crude analyte thin-films and (**f**) covered with different random ellipse particles. (**g**–**i**) The summaries of the relationships between the frequency shifts and refraction indexes of (**d**–**f**).

In consideration of the realistic materials, including rough PDMS, crude analyte thin-films and irregular biological cells, three more realistic models were simulated to imitate real situations. The schematics of the top views of SSM on a rough PDMS substrate, covered with crude analyte thin-film and covered with random ellipse particles are shown in Figure 8a–c. The roughness of the PDMS surface and the analyte thin-film was introduced by texturing surfaces, with the maximum texturing depth determined to be 200 nm. The random ellipse particles were introduced by determining three ellipse radii haphazardly in the range of 3 to 5 μm. Figure 8d shows the transmission spectra of SSM on rough PDMS substrates with increasing *n_b_* values. The resonances are red-shifted significantly compared to those results in Figure 4a. This implies that the sensing capacity of SSM can be enhanced by texturing the substrate surface. The relationships between frequency shifts and *n_b_* values are summarized in Figure 8g. The sensitivities are 302 GHz/RIU for Q mode, 414 GHz/RIU for O mode and 502 GHz/RIU for H mode, which are greater than those results in Figure 4b (286, 390 and 460 GHz/RIU for Q, O H modes, respectively). These enhancements are due to the additional space below the metallic SRRs after texturing the PDMS surface. Figure 8e shows the transmission spectra of SSM covered with crude analyte thin-films with different *n_c_* values. The sensitivities of Q, O and H modes are 26, 36 and 49 GHz/RIU, respectively, which are lower than those in Figure 5a. Figure 8f shows the transmission spectra of SSM covered with random ellipse particles with different *n_c_* values. The sensitivities are 103, 158 and 258 GHz/RIU for Q, O and H modes, respectively. These values are lower than those in Figure 6b. As mentioned above, the sensitivity could be improved by increasing the hemisphere number and channel width of the microfluidic chip. This proves that the results of the lower sensitivities in Figure 8h,i, caused by the rough analyte thin-film and the deformation of ellipse particles owing to the coupling effects, are minor for SSM covered with a crude analyte thin-film and random ellipse particles.

## 4. Conclusions

In conclusion, an SSM-based biochemical sensor composed of centrosymmetric Au SRRs on a PDMS substrate was proposed and investigated in the terahertz wavelength. By changing different surrounding dielectric environments, the sensing performance of the proposed biosensor was verified. The results show that the increments of environmental refraction indexes (*n_b_*, *n_a_*, *n_c_* and *n_s_*) lead to the apparent red-shifts of resonances in the terahertz frequency range. Furthermore, the higher plasmon mode exhibits larger frequency shifts and greater sensitivity in biosensing applications. The dielectric hemispheres and microfluidic channels are further integrated into the proposed SSM device to verify the biosensing abilities of SSM. This indicates that the increments of hemisphere concentration or microfluidic channel width are beneficial for improving the sensing performance of an SSM-based biosensor. The highest sensitivity was 471 GHz/RIU. Finally, we propose three more realistic models to more reasonably mimic a real sensing situation, and the corresponding highest sensitivity was 502 GHz/RIU. This proposed SSM is quite suitable for use in biochemical and biomedical sensing applications.
